# Microgravity simulation by diamagnetic levitation: effects of a strong gradient magnetic field on the transcriptional profile of *Drosophila melanogaster*

**DOI:** 10.1186/1471-2164-13-52

**Published:** 2012-02-01

**Authors:** Raul Herranz, Oliver J Larkin, Camelia E Dijkstra, Richard JA Hill, Paul Anthony, Michael R Davey, Laurence Eaves, Jack JWA van Loon, F Javier Medina, Roberto Marco

**Affiliations:** 1 Centro de Investigaciones Biológicas (CSIC), Ramiro de Maeztu 9, E-28040, Madrid, Spain; 2 Departamento de Bioquímica, Universidad Autónoma de Madrid (UAM), Arzobispo Morcillo s/n, E-28029, Madrid, Spain; 3 School of Biosciences, University of Nottingham, Sutton Bonington Campus, Loughborough, LE12 5RD, UK; 4 School of Physics & Astronomy, University of Nottingham, Nottingham NG7 2RD, UK; 5 Dutch Experiment Support Center, DESC at OCB-ACTA, VU-University and University of Amsterdam, Amsterdam, the Netherlands

## Abstract

**Background:**

Many biological systems respond to the presence or absence of gravity. Since experiments performed in space are expensive and can only be undertaken infrequently, Earth-based simulation techniques are used to investigate the biological response to weightlessness. A high gradient magnetic field can be used to levitate a biological organism so that its net weight is zero.

**Results:**

We have used a superconducting magnet to assess the effect of diamagnetic levitation on the fruit fly *D. melanogaster *in levitation experiments that proceeded for up to 22 consecutive days. We have compared the results with those of similar experiments performed in another paradigm for microgravity simulation, the Random Positioning Machine (RPM). We observed a delay in the development of the fruit flies from embryo to adult. Microarray analysis indicated changes in overall gene expression of imagoes that developed from larvae under diamagnetic levitation, and also under simulated hypergravity conditions. Significant changes were observed in the expression of immune-, stress-, and temperature-response genes. For example, several heat shock proteins were affected. We also found that a strong magnetic field, of 16.5 Tesla, had a significant effect on the expression of these genes, independent of the effects associated with magnetically-induced levitation and hypergravity.

**Conclusions:**

Diamagnetic levitation can be used to simulate an altered effective gravity environment in which gene expression is tuned differentially in diverse *Drosophila melanogaster *populations including those of different age and gender. Exposure to the magnetic field *per se *induced similar, but weaker, changes in gene expression.

## Background

Since the beginning of life on Earth, organisms have developed under the influence of Earth's gravity. Evolution has provided a number of different solutions to the mechanical challenge of supporting the weight of a living organism [1-4]. In general, the mechanical stresses induced by gravity on an organism increase with its mass, although for organisms living in water, the effect of gravity is to some extent mitigated by buoyancy. Gravity has an important effect on the development of seedlings and studies show that the gravitational sense mechanism acts at the cellular level (geotropism) [5]. Another well-known effect of altered gravity on living organisms is the reduction of the strength of the bones of astronauts after they have undertaken long missions in orbiting spacecraft. The gravitational acceleration, which is *g *= 9.8 ms^-2 ^on the Earth's surface, exerts a force of 9.8 N on a mass of 1 kg. The reduced gravity on the surfaces of Mars (0.37 *g*), the Moon (0.18 *g*), and the microgravity conditions in orbiting space stations may have important effects on astronauts manning the first space colonies, and on the development of animals and plants. It is also possible that zero- and reduced-gravity influences the behaviour of micro-organisms, either directly or through the effect of reduced gravity on the environment, *e.g*. modified convection in fluids and gases could affect bacterial physiology [6].

One of the current challenges is to advance our understanding of the way genomic information is modulated by different physical and environmental forces to produce the diverse phenotypes that are encountered in biology. The fruit fly *Drosophila *is an ideal organism with which to explore environmental effects on the genome; the genome of more than twelve species of *Drosophila *have had their genomes sequenced and are available in Flybase [7] in different gene annotations.

Several methods exist to simulate "weightlessness" on Earth, such as the Random Positioning Machine (RPM) [8,9]. Another approach to study the response of organisms to changes in gravity is the use of diamagnetic levitation (See, for example, [10-14]). Diamagnetic material, which includes water and biological tissue, is repelled from magnetic fields. Within the bore of a powerful 'Bitter' electromagnet or superconducting solenoid, the repulsive diamagnetic force on water can be enough to balance the weight of the water so that it levitates [15-18]. Most soft biological tissues can be levitated under the same conditions, since water is the main constituent of the tissue by mass, and most of the remaining material has a magnetic susceptibility and a density similar to that of water [19]. This technique differs from floatation, in that the diamagnetic force acts throughout the body of the levitating object, at the molecular level, not just at its surface, as is the case in buoyancy. In this respect, the diamagnetic force can be compared to the centrifugal force that balances the force of gravity on an object orbiting the Earth or another planet.

Several of the authors have experience in testing the effects of gravity using space laboratories [20-22] and also in ground simulation facilities, such as the RPM [23-25]. In the fruit fly, *Drosophila*, extensive gene expression changes, as well as changes in the motility behaviour of imagoes, occur in both real and simulated microgravity. Conversely, hypergravity (up to 10 g) has a relatively weak effect on motility and gene expression. These changes can affect additional traits, such as the life-span of the flies [26]. In addition, differences have been observed in the motility and behaviour of the flies exposed to real or simulated microgravity [20,26] including the behaviour of levitating flies in a superconducting magnet [27].

In this paper, we investigated the effect of levitation (0 g*) and simulated hypergravity (twice Earth-gravity, 2 g*) on the fruit fly *Drosophila melanogaster *using a specially-designed superconducting magnet with a closed-cycle cryogenic system at the University of Nottingham (Additional file 1, Figure S1). We levitated the flies in continuous experiments of up to 22-days duration. Effects were observed on the overall gene expression patterns, and also a significant delay in the development of the fruit flies from embryo to adult, compared with control conditions in Earth's gravity. The results were compared with those of similar experiments in the RPM. We also studied the effect on the flies of a spatially uniform strong magnetic field (16.5 Tesla), in which the diamagnetic forces were negligible so that flies experienced normal gravity.

## Methods

### Levitation magnet and experimental arrangement

The superconducting magnet used to levitate the flies has a 5-cm diameter vertical bore, which is temperature-regulated by forced air flow through the bore (Additional file 1, Figure S1). The magnetic field is strongest at the centre of the bore. The force on diamagnetic material in the magnetic field is proportional to the product of the field strength, measured in Tesla (T), and the field gradient, measured in Tm^-1^. The expression for the effective gravity (g*) as a function of magnetic field is given in Additional file 1[28]. The field gradient is zero at the centre of the bore, and rises to a maximum 85 mm above and below the centre.

**Figure 1:**
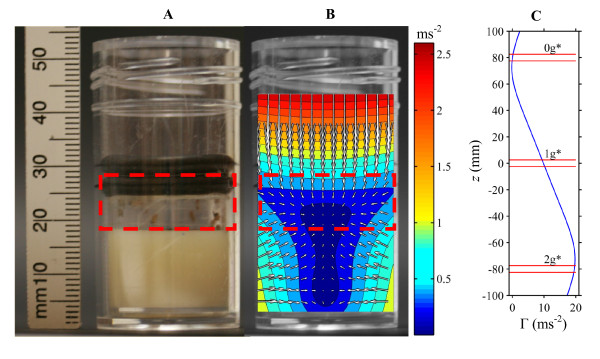
**Effective gravity along the magnet bore and arena container properties**. **A) **Side view of an arena contained within a transparent plastic tube. The flies were constrained to the volume indicated by the red rectangle, between a cellophane disc (retained by two black o-rings visible in the picture) and a semi-solid culture medium (off-white material at bottom of tube). **B) **Effective gravity acting on water within the 0 g* tube. The colour indicates the magnitude of the effective gravity (ms^-2^). Arrows show the magnitude and direction of the effective gravity. The residual gravity on the flies (within the red rectangle) is less than 5 × 10^-2 ^g. **C) **Effective gravity acting on water on the solenoid axis, Γ, as a function of vertical position, *z*. The centre of the solenoid is at *z *= 0 mm. The arenas were placed between the pairs of horizontal red lines shown on the plot.

When the field at the centre of the solenoid is 16.5 T, the diamagnetic force on a sample of liquid water placed approximately 80 mm above the centre is sufficient to balance the force of gravity, so that the water levitates as a freely-suspended droplet (Figure 1 and Additional file 1, Figure S2). At the levitation point, the effective gravity acting on the liquid is zero. The weight of the same sample of water placed approximately 80 mm below the centre of the solenoid is twice that outside the magnet; in this sense, the effective gravity acting on the water is twice Earth's gravity (2 *g*).

Stable, containerless levitation of water and biological material is possible using this magnet, but for a biological specimen such as *Drosophila*, performing containerless experiments is not practical, since it is impossible to perform equivalent controls. Instead, the flies were contained in three 25 mm-diameter, 10 mm-tall 'arenas' constructed within 25 ml clear plastic tubes (Figure 1 and Additional file 1, Figure S3). The tubes were placed to enclose the stable levitation point, the centre of the solenoid where the magnetic field was strongest and the 2 *g *point of water respectively. For convenience we label the three arenas, from top to bottom, 0 g*, 1 g* and 2 g*. The asterisk (*) indicates that the '*g*-value' (0 *g*, 1 *g*, 2 *g*) refers to the effective gravity on *water *at the centre of each arena. It also serves as a reminder that there was a strong magnetic field present in these arenas. The magnetic field was 11.5 T at the centre of the 0 g* and 2 g* arenas and 16.5 T at the centre of the 1 g* arena. Note that, in using the label "0 g*" to refer to the upper arena in the magnet bore, we do *not *mean that the effective gravity acting on liquid water within the 0 g* arena was precisely zero everywhere. In fact, the effective gravity acting on water varied by a few percent within all three arenas in the magnet bore, owing to the spatial variation of the magnetic field. The effective gravity acting on water inside the 0 g* tube was zero at the levitation point, rising to approximately 5 × 10^-2 ^*g *at the walls of the arena (Figure 1C and Additional file 1, Figure S3). We use the term 0 g* only as a convenient label for the arena and the tube in which it was contained.

A fourth arena, labelled 1 *g *(i.e. without *), was placed in an incubator well away from the solenoid, where the magnetic field of the solenoid is small compared to the Earth's magnetic field. Video images of the flies in each container were recorded using CCD cameras; white LED lighting on a 12 hour photoperiod cycle provided illumination for the videos.

There have been reports that static high magnetic fields (several Tesla fields with no levitation) can produce effects on bacteria [29], plants [30,31], mammals [32], flies [33] and the development of frog eggs [34]. By performing experiments simultaneously at 0 g*, 1 g* and 2 g* and in the control outside the magnet (1 *g*), we were able to distinguish unambiguously between the effects on the flies of altering their net effective weight, and any other effects of the high magnetic field.

### Biological experiments

To maximise the output of our magnet experimental time we performed three experiments under different environmental conditions inside the magnet, which, for convenience, we refer to by the duration of the experiment ('short-term', 'medium-term', 'long-term').

#### 1) "Short-term" (1 day) experiment

Virgin females were mated overnight with an excess of young males. Male and female 1-2 day old Oregon R *Drosophila melanogaster *flies were both exposed to diamagnetic levitation for the following 26 hours at 14°C. During this time, their behaviour was monitored using CCD cameras. After exposure to the magnetic field, the eggs laid inside the magnet were counted and were allowed to hatch in order to monitor the normality of the developmental process of eggs formed during levitation conditions. Both males and females were homogenised separately to study their gene expression profiles by microarray analysis. Our choice of 14°C for the bore temperature was based on preliminary observations indicating that any effects may be amplified by suboptimal growth conditions [21]. For comparison, we performed an additional control well away from the magnet (i.e. far enough away that it was not influenced by the field of the superconducting magnet), but otherwise under similar conditions.

#### 2) "Medium-term" (5 day) experiment

Pupae (OregonR *Drosophila *early pupae) collected in Madrid and transported to Nottingham at 14°C were incubated in the magnet for 5 days at 24°C, to analyse the effect on the metamorphosis of the flies replicating the design of a real space experiment performed on-board the ISS [21]. Pupae were allowed to develop into adults inside the magnet to study phenotypic alterations. Recently-hatched flies were separated into males and females and then homogenised in bulk. RNA was extracted and assayed using microarrays to determine the effect of the magnetic field on the expression profile.

#### 3) "Long-term" (22 day) experiment

The samples (recently-laid OregonR *Drosophila *eggs) were exposed to the magnetic field for 22 days at 19°C to analyse the effect of the magnetic field and altered effective net weight of the flies on their entire life cycle, from embryo to embryo. The larvae were allowed to pupate *in situ *and a second generation of flies hatched under these conditions. The second generation imagoes were removed from the magnet and separated into males and females. The males were homogenised to extract the RNA and assayed for gene expression using microarrays. The females were allowed to lay eggs in order to monitor embryo development. In addition to the 19°C experiments, we also performed an additional external control (*i.e*. outside the magnet) at 24°C, and a replicate long-term experiment (*i.e*. 22 days at 19°C) in the RPM (0 *g*^RPM^, 1 *g*^RPM^) at the Dutch Experiment Support Center, DESC at OCB-ACTA, VU-University of Amsterdam, the Netherlands.

Table 1 summarises the experimental design of the short-term (1 day), medium-term (5 days) and long-term (22 days) experiments. The 54 hybridised RNA samples were identified with a CEL file name and classified by experimental duration, gender and environmental conditions (g* force, magnetic field, temperature). This microarray dataset has been published in the MIAME compliant Array Express Archive [EMBL_EBI:E-MEXP-2082].

**Table 1:** Description of the 54 microarray samples used in this paper

Experimental design	Gender	Ground Based Facility	Condition (effective force, g*)	Name of CEL file replicates			
**Short-term**		Magnet	0 g*	S0A	S0B	S0C	
		
(26 h/14°C)		Magnet	1 g*	S1A	S1B	S1C	
		
**Exposure:**	Females	Magnet	2 g*	S2A	S2B	S2C	
		
From 1-2 days to		---	1 *g *(14°C)	SCA	SCB	SCC	
		
2-3 days imagoes		---	1 *g *(24°C)	SDA	SDB	SDC	
	
		Magnet	0 g*	X0A	X0B	X0C	
		
		Magnet	1 g*	X1A	X1B	---	
		
	Males	Magnet	2 g*	X2A	X2B	X2C	
		
		---	1 *g *(14°C)	XCA	XCB	---	
		
		---	1 *g *(24°C)	XDA	XDB	---	

**Medium-term**		*Magnet*	0 g*	*M0A^#^*	*---*	*---*	
		
(5d/24°C)	*Females*	*Magnet*	1 g*	*M1A^#^*	*---*	*---*	
		
**Exposure:**		*---*	*1 g*	*MCA^#^*	*---*	*---*	
	
From early pupae		Magnet	0 g*	N0A	N0B	---	
		
to recently	Males	Magnet	1 g*	N1A	N1B	N1C	
		
hatched imagoes		---	1 *g*	NCA	NCB	NCC	

**Long-term**		Magnet	0 g*	L0A	L0B	L0C	L0D
		
(22d/19°C)		Magnet	1 g*	L1A	L1B	L1C	
		
**Exposure:**	Males	*Magnet*	2 g*	*L2A^#^*	*---*	*---*	
		
From embryo to		---	1 *g*	LCA	LCB	LCC	
		
mature imagoes		RPM	0 *g*^RPM^	R0A	R0B	---	
		---	1 *g*^RPM^	RCA	RCB	RCC	

From the group of 54 samples, 50 have been used in the main analysis, while four (not replicated, *orphan^#^*, microarrays shown dashed in the table) have been used only for comparisons or as internal controls. Submission to the MIAME compliant Array Express Archive at EMBL_EBI has been done [EMBL_EBI:E-MEXP-2082].

### Gene expression analysis using the *Drosophila *microarray

Total RNA extracted from fruit flies was hybridised using Affymetrix^® ^Drosophila 2.0 whole genome GeneChip arrays. We analysed two to four independent biological replicates of the experiments including RNA from ten flies per array; the number of arrays in each condition is indicated in Table 1. Only one array was hybridised for females from the medium-term experiment and for male flies from the 2 g* tubes in the long-term experiment, owing to reduced quality of these samples' replicates. Differentially-expressed genes were detected using a Volcano-plot comparison with GeneSpring GX 10 software (version 2.1), with a p-value cut-off of 0.05 (except in the four single-array conditions highlighted in italics on Table 1) and a fold difference > 1.7 including a parametric test that assumed equal variances.

A global and integrative analysis using "gene expression dynamics inspector" (GEDI) self-organising maps, was performed using the above software [35]. Firstly, we applied the Robust Multichip Average (RMA) algorithm for background correction, normalisation and expression-level summarisation of the arrays (see above). Second, we identified 18921 probe sets that showed an expression above the 20^th ^percentile, relative to the 1 *g *control, in half of the experimental conditions. For these sets, we calculated the average signal ratio, and used this value for GEDI analysis automatic clustering. Mosaics of 20 × 16 grid size (average of 59 genes/tile) were obtained for each condition using the standard settings of the software (more methodological information can be found in Additional file 1).

## Results

### A) Diamagnetic levitation of *Drosophila melanogaster*

In the 0 g* tube, we observed flies levitating freely (i.e. not in contact with any surface, or flying) within 1-2 mm of the levitation point of liquid water (Additional file 2, and [27]). This is not unexpected because the flies have a high water content. The net effective weight of a freely levitating fly is zero, in the sense that there is no net (gravitational plus magnetic) force on the fly. Although we observed a few flies levitating freely, the majority remained in contact with the walls, floor and ceiling of the arena enclosed within the tube.

The net effective weight of an individual fly on the walls, floor or ceiling depends on its position within the arena (Figure 1C). The effective weight is less than 5% of its weight outside the magnet throughout the arena.

We observed that the levitation position of the freely levitating flies varied with their hydration. Dehydrated (dead) flies levitated a few millimetres lower in the magnet than living flies. There was a 1-2 mm difference in levitation position between each of the *Drosophila *stages. This is consistent with the greater water content of the embryos and early larvae (more than 80% of total mass), reaching the lower level at late pupae (less than 70% of total mass) in comparison with an average 75% of water content in adults [36,37].

### B) Delay of development due to the magnetic field

The results of the "long-term" (22-day) experiments demonstrated that 1-12 hour old embryos can develop fully, progressing from larvae to pupae to imagoes, both in the RPM and in a strong magnetic field up to 16.5 T. Development in the magnet (0 g*, 1 g* and 2 g*) was slightly but reproducibly delayed by one day, compared to the 1 *g *control outside the magnet, suggesting that metamorphosis can be delayed in one or more developmental checkpoints. A less evident delay in development was observed in the RPM.

Table 2 shows the number of flies that developed from eggs laid in the magnetic field in a "short term" (1-day) experiment. After the females were mated, 25 males and 25 females were selected randomly and placed together in the same container in the magnet for 26 hours at 14°C. The eggs laid during those 26 hours were incubated outside the magnet and the flies that developed from the eggs were counted. The results show that exposure to the strong magnetic field during oogenesis and laying caused a large reduction in the number of adult flies that developed from the eggs. The number of flies that developed from eggs laid in the 16.5 T magnetic field (1 g* tube) was just 31% of the number that developed from eggs laid in the 1 g control tube outside the magnet. In the 0 g* and 2 g* tubes, where there was a significant field gradient, the reduction in the number of flies was even greater, being only 5-6% of the number resulting from the 1 g control.

**Table 2:** Imagoes developed from eggs laid during the medium term experiment

	Control at 24°	Control at 14°C	1 g* 14°C	0 g* 14°C	2 g* 14°C
Total number of flies	152	86	27	5	4

% of flies in relation to 14°C	177%	100%	31%	6%	5%

% of flies in relation to 24°C	100%	57%	18%	3%	3%

25 females and 25 males were exposed to the magnetic field for 26 hours continuously. Prior to this, the males and females had been kept together for 24-48 hours. The table shows the number of eggs, deposited by the females whilst *inside *the magnet (0 g*, 1 g*, 2 g*), that went on to develop into imagoes *outside *the magnet. Two controls, one at 24°C and one at 14°C outside the magnet were performed.

### C) Magnetic field affects gene expression

**Figure 2:**
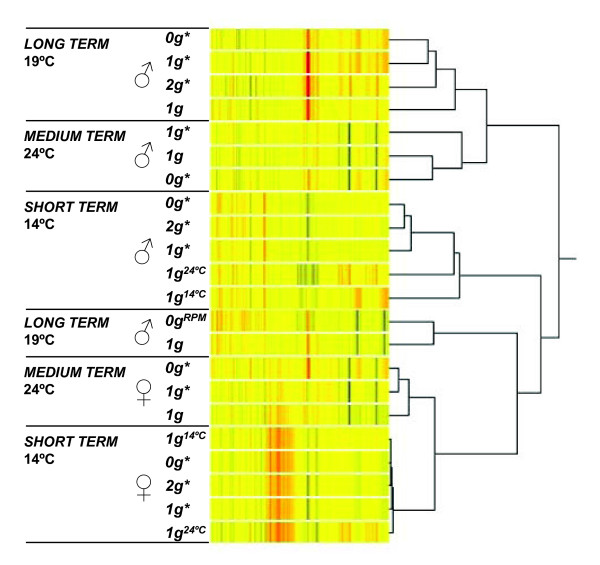
**Clustering of the 22 analysed transcriptional profiles**. Clustering of the transcriptional profiles is revealed by a condition tree calculated with a hierarchical cluster algorithm, using Pearson absolute distance metric and the average linkage rule. The length of the branch is an indicator of the number of gene expression variations found between each condition and experiment (shorter distances indicate a greater resemblance).

Using Affymetrix whole genome microarrays (Drosophila version 2.0 with 18952 probesets and GeneSpring GX), we analysed the gene expression profile of *Drosophila *exposed to the magnetic field, and compared the results with *Drosophila *in a temperature-controlled incubator placed well away from the magnet (1 *g*). We also compared the results of "short-term", "medium-term" and "long-term" experiments. In most experiments we performed three replicates, except in a small number of cases (identified by dashes in Table 1) where we were unable to obtain one or more replicates owing to random contamination of the extractions, or time constraints on the use of the magnet. We also compared microarray results from the "long-term" magnet experiment with 5 microarrays from a RPM microgravity simulation experiment. The transcriptional profiles of each experiment are shown in Figure 2. Here, the profiles have been presented as a "condition tree", in which similarities between transcriptional profiles are reflected in the grouping of the experiments within the tree. For example, the transcriptional profiles from the 0 g*, 1 g* and 2 g* tubes in the "long-term" experiment display readily identifiable similarities and so are grouped together in the diagram. Likewise, common features can also be identified in the profiles from the "medium-term" and "short term" experiments. There were significant differences in the profiles from experiments of different duration. For example, there were clear differences between the profile of flies in a 0 g* tube in a long-term experiment and the profile of flies in a 0 g* tube in a short-term experiment. The variation between experiments of different durations was smaller for females than for males. These results showed that the precise biological state of the organism (*i.e*. age, gender, temperature) was important in the magnetic field effect.

The Venn diagram in Additional file 1, Figure S4 shows the number of genes that changed their expression levels in the magnet compared with the 1 *g *external controls located well away from the magnet. The *number *of genes affected was nearly independent of the duration of the experiment, or the location (0 g*, 1 g* or 2 g* arena) inside the magnet. Interestingly, the group of genes affected in the long-term experiment was different from the group affected by the medium-term experiment. Similarly, the group of genes affected by the short-term experiment was different from both the long-term and medium term experiments, although there was some overlap in the three groups of genes affected. We identified 496 genes that were sensitive to the strong magnetic field in males, *i.e*. genes that are up- or down-regulated in one or more of the long-, medium- and short-term experiments in the magnetic field. Of this group of 496 genes, 105 were common to two different experiments. We found only one gene common to all three experiments (long, medium and short-term), namely CG33070-RB, Sex Lethal, encoding an RNA binding protein.

In females, 474 genes were sensitive to the magnetic field, of which 115 were common to flies in two different experiments. Fourteen genes were common to flies in all three experiments; three of these genes have been annotated as heat shock proteins.

Less than 10% of the magnetic field-sensitive genes were common to both males and females; 5 were observed in two or more experiments, with 47 observed in one or more experiments.

We also analysed the short-term, medium-term and long-term experiments separately, in order to identify the differentially-expressed genes induced by the magnetic field. The *number *of genes sensitive to the magnetic field in the individual experiments is shown in a series of Venn diagrams in Additional file 1, Figure S4.

### D) Isolating the effect of magnetically-altered effective weight from other effects of the strong magnetic field

The above results indicate that the strong magnetic field present in all three tubes (0 g*, 1 g* and 2 g*) had a significant effect on gene expression of the flies. In order to locate genes that could play a role in gravisensing or adaption, it was necessary to account for the effects of the strong magnetic field. We attempted to isolate the effect of the vertical diamagnetic force on flies (which alters the effective net weight of the flies), from any other effects of the magnetic field by comparing the gene response of the flies in the 0 g* and 2 g* tubes to those in the 1 g* tube

Two different approaches were used:

• Approach 1: A list of genes that were up- or down-regulated in the 0 g* tube in the magnet was compiled and compared with the external control outside the magnet (1 *g*). We repeated this for the 1 g* and 2 g* tubes, and then removed from the 0 g* vs 1 *g *and 2 g* vs 1 *g *lists those genes that appeared in the 1 g* vs 1 *g *list.

This approach was based on the results in Additional file 1, Figure S4, first and second rows, in which it is evident that few genes were common to flies in all tubes in the magnet (0 g*, 1 g*, 2 g*) and the 1 *g *control tube. We found that between 20% and 50% of the genes affected by a change in effective gravity (0 g* or 2 g*) were present in flies in both 0 g* and 2 g* tubes.

• Approach 2: We compiled two lists of genes: one of genes that were up- or down-regulated in 0 g* compared with 1 g*, and one of genes that were altered in 2 g* compared with 1 g*.

In both approaches, we made the prior assumption that the magnetic field effects observed in the 1 g* tube (at 16.5 T) had nearly the same influence on the genes in the 0 g* and 2 g* tubes where the field was smaller (11.5 T).

The numbers of genes in the lists resulting from the two procedures described are shown in the highlighted third row of Additional file 1, Figure S5. The results from the RPM experiment (described in the Methods section) are also shown for comparison. The five gene ontology groups with the highest statistical significance in each gene list are in the table in Additional file 1, Figure S5. Most of the individual genes affected were not the same in the different tubes (0 g*, 1 g*, 2 g*). However, we could identify a common theme in the affected gene *groups*, which consisted of defence/immune/stress response and cell signalling gene ontology (GO) groups.

**Figure 3:**
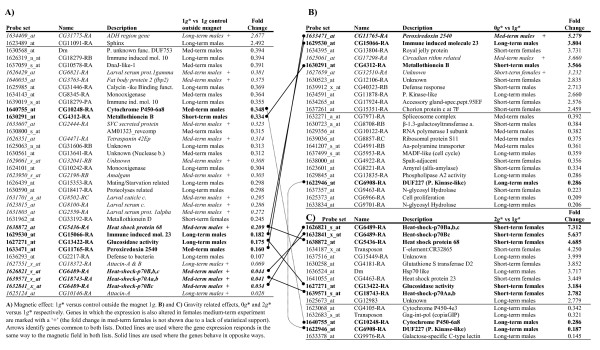
**List of genes that respond to the magnetic field with a fold increase of more than 2.5 or fold decrease of more than 0.4**.

Figure 3A lists those genes with an increase in expression of 2.5 fold or more, or decrease of 0.4 fold or more, at 1 g*, compared to the 1 *g *control (at 0 T). Those genes with the largest change in expression are listed towards the top of the table. In Figure 3B, we list genes that have a similar significant change in expression at 0 g*, compared to 1 g*. Figure 3C shows the same list for the change in gene expression at 2 g* compared to 1 g*. The genes listed in Figure 3B and 3C are those which were affected by the vertical diamagnetic force alone, and not by any other effects of the magnetic field. Since the vertical diamagnetic force altered the effective net weight of the flies, these genes may have a role in gravisensing or altered gravity adaption.

There was only one gene with a similarly significant change in expression in the RPM experiment (an unknown function gene, CG15065 with a 0.365 fold expression change in the RPM). Some of the groups of genes identified in section C appear again in the lists shown in Figure 3. Of particular interest are genes that appear in more than one experiment (for instance, those in italics that are also present in the medium-term experiment on females, orphan arrays) owing to their additional statistical significance.

As shown in Figure 3A, there were only two genes (of unknown function) that were significantly up-regulated at 1 g* compared to the 1 *g *control. Of those that were significantly down-regulated, the types of genes that appeared most frequently were those related with heat shock, immune/defence response, oxidation and lipid processes. It is interesting that the same heat shock genes that appear in this list also appeared in the 2 g* vs 1 g* list (Figure 3C), but in the latter they were over-expressed; the "Heat-shock-p70" gene was severely down-regulated in 1 g* compared to 1 *g *in males in a "medium-term" experiment, and up-regulated in 2 g* compared to 1 g* in females in a "short-term" experiment. None of the heat-shock genes that appeared in Figure 3A appeared in the 0 g* vs 1 g* list (Figure 3B), but two immune response genes (Peroxiredoxin 2540 and Immune Induced molecule 23) were common to both of them, being down-regulated in 1 g* and up regulated in 0 g* experiments.

The sensitivity of these genes to the magnetic field was made more evident when we integrated these genes into a virtual cellular pathway. We used Pathway Studio 6.01 for a graphical output of the relations among the genes in each group. Additional file 1, Figure S6 shows the magnet affected genes (Figure 3A), including some connector genes in order to fill the gaps amongst them. From the pathway, most of the genes were linked in less than two steps with other affected genes, suggesting that their functions were connected in the cell.

Many of the genes listed in Figure 3 have unknown function, and it would be revealing to identify the function of each of these genes. One of these unknown-function genes deserves special attention. CG6908 appeared in both 0 g* vs. 1 g* and 2 g* vs. 1 g* lists (Figure 3B &3C), and was strongly repressed (4 to 5 fold) in both conditions. Gene CG6908 encodes a relatively large protein with a PKC-like domain in the middle and was not changed due to magnetic field (Figure 3A). Therefore, this gene seems specifically sensitive to the magnetic field gradient (net gravity change).

We assessed how many of these magnetically-affected genes were also present in the group of 36 genes that were up- or down-regulated in the RPM long-term experiments. This result is shown in the grey box in Additional file 1, Figure S4. We found that only one gene was up- or down-regulated in both the RPM and the "long-term" magnet experiments, compared to the relevant control (CG32641-RA; GenBank accession number). This gene encodes a protein with Heat shock protein/Chaperone DNAJ domains. Curiously, this gene was altered only at 1 g* in the magnet, but not at 0 g* tube, as one might expect. When we compared the group of 36 RPM-altered genes with those altered in any of the short-term experiments in the magnet, we found that three genes were commonly affected. One of these was "Yuri Gagarin", a gene selected previously as one of the gravity-response genes [38]. The other two, automatically annotated unnamed genes, remain to be analysed further.

### E) Global transcriptional states in the magnet: GEDI analysis

**Figure 4:**
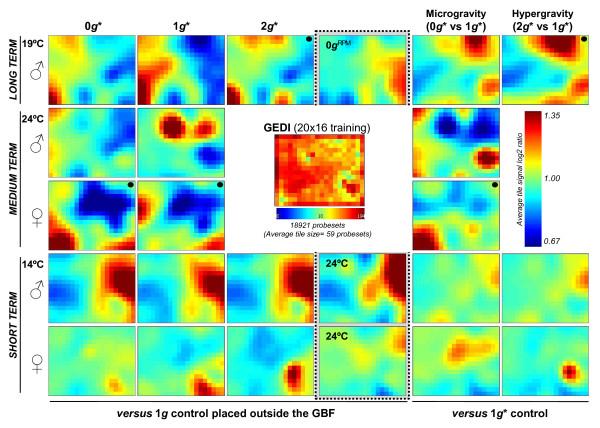
**GEDI 20 × 16 clustering analysis based on the three magnet experiments and one experiment at RPM**. One experiment per row is shown for separate male and female data, in different Ground Based Facilities (GBF). The colour scale on the right indicates the average log to the base 2 ratios of each cluster compared to the parallel 1 *g *control for conditions 0 g*, 1 g*, 2 g* and others (first four columns), and versus the 1 g* control in the fifth (0 g* vs 1 g*) and sixth (2 g* vs 1 g*) columns. The centre panel indicates the number of probesets (18921 probesets) included in each cluster (20 × 16 clusters with an average size of 59 probesets per cluster). Panels obtained from an orphan array are indicated by a black dot. Panels not linked to the magnetic field effect (*i.e*. those in the fourth column) are enclosed by a dashed line.

Taking into account that there are very few individual genes that were affected consistently between short-, medium- and long-term experiments, we analysed the transcriptome status as a whole. We analysed the microarray data with the "Gene Expression Dynamics Inspector" (GEDI) program [35]. The GEDI software organises the gene expression patterns into mosaics of *n *x *m *tiles. Each tile corresponds to a cluster of genes that behave similarly across conditions, designated a centroid. Different colours reflect the expression intensity of a centroid in each condition (in our case the average ratio of intensities compared to 1 *g *controls). Additionally, GEDI places similar centroids close to each other in the mosaic, creating an image of the transcriptome and allowing its analysis as an entity by simple visual comparison of the mosaics corresponding to different conditions. For this analysis, we normalised the expression data as indicated in the supplementary methods (Additional file 1). We used 18921 probe-sets for the GEDI analysis. They were placed in 20 × 16 mosaics with an average of 59 genes per centroid. The results obtained are available as GEDI original files in Additional file 3 and summarised in Figure 4.

As expected from Results sections C and D, the transcriptome obtained from the 0 g* tubes responded differently in each of the experiments (short-, medium- and long-term), and the response in 0 g* (compared to 1 *g *and 1 g* controls) depended on the sex of the flies. In all cases, the response to the magnetic field was weak; most of the genes showed a log to the base 2 ratio change in expression of < 1.35 versus the control expression, as indicated by the colour scale (Figure 4). We observed a similarly weak response in the other two tubes (1 g*, 2 g*) in the magnet. We note that the 0 g* and 2 g* images seem to be more closely related to each other than to the 1 g* image. A RPM produced an effect superficially similar to that of the magnetic field (in the 1 g* arena), in that the number of genes affected and the expression change of these genes were similar to that observed in the magnet, but the RPM affected different gene clusters (Additional file 1, Figure S4 and S5). A change in temperature to 24°C produced a similar effect. However, the sensitivity of the transcriptome to the external physical parameter change (magnetic field, field gradient, temperature, RPM movements) depended on the biological state of the sample. For example, female flies seem to be less sensitive to short-term exposure to strong magnetic fields than males (the medium-term experiment had the opposite trend, but this could be due to the fact that the medium-term experiment with females was carried out with insufficient replicates due to time constraints). If we compare male and female patterns in the 1 g* tube of the medium-term experiment (in Additional file 1, Figure S4), it is possible to localise a group of genes that are up-regulated by the magnetic field in males, but down-regulated in females, suggesting differential transcriptome adaption responses to stress in selected populations of the same species.

Interestingly, the last two columns on the right of Figure 4, showing the 0 g* vs. 1 g* response and 2 g* vs. 1 g* response do not show opposite trends in gene expression, as one might expect initially. This is especially clear in experiments with male flies that produced very similar transcriptome images in the GEDI analyses. An opposite trend has been observed in mechanical simulators, RPM vs. centrifuge [21].

## Discussion

### A) Development and behaviour is modified by high gradient magnetic fields

We have observed both embryonic development and pupal metamorphosis under diamagnetic levitation conditions (short- and medium-term experiments). Additionally, we observed a complete *Drosophila *life cycle, from embryo to embryo, in a magnetic field of up to 16.5 T, over a 22 day long term experiment. In this experiment, the number and rate of eggs laid could not be evaluated due to the lack of *in situ *imaging. In a later, short-term experiment, we observed that the early stages of embryogenesis were affected by the strong magnetic fields present in the magnet bore (a one third decrease in fertility in the 1 g* tube, Table 2).

The observation of a decrease in the number of flies resulting from eggs laid in the 1 g* tube, but a *greater decrease *in flies resulting from eggs laid in the 0 g* and 2 g* tubes, indicates that *both *magnetic field and the field gradient played a role in this effect. We emphasise that the magnetic field in the 0 g* and 2 g* tubes is less than in the 1 g* tube; the field in these two tubes is 11.5 T, compared to 16.5 T in the 1 g* tube. The large field gradient (~100 Tm^-1^) in the 0 g* and 2 g* tubes (absent in the 1 g* tube) altered the effective net weight of the flies, which affected their motility, as reported in [27]. A magnetic field in the absence of a field gradient (*i.e*. the conditions in the 1 g* tube) can also affect the organism, for example through magnetic alignment of biostructures in the magnetic field (see, for example,[34,39-42]), or through induction of an electromotive force (by Faraday's law) as the organism moves through the strong magnetic field [39].

It is important to point out here that behavioural changes, delays in development, and altered rate of oviposition have been described in several experiments in orbiting spacecraft with *Drosophila *[43-46]. The final conclusion of those experiments was that normal development is possible in "space" [43-46], despite the microgravity effects. Consistently, in our long term experiments some flies were able to develop in each magnet condition although their development was even slower than in the RPM or in real space mission experiments, so the effects observed in the short term experiment were not completely deleterious. Changes in the motility of diamagnetically levitated flies [27] could be an explanation for a change in the oviposition rate; embryo retention is a typical behavioural response to environmental stress. This could explain the reduced number of imagoes that developed from eggs laid in the magnetic field, and in particular, at 0 g* and 2 g*.

### B) Global transcriptional effects are observed indicating that magnetic field affects some external stress response elements

Microarray analysis of the RNAs indicated that there were changes in the gene expression of imagoes that were exposed transiently to, or developed during metamorphosis in the presence of a diamagnetic force (0 g* and 2 g* tubes), and/or a high magnetic field (Figure 4). The main conclusions from the gene expression analysis are that the exposure of the samples to the strong magnetic field, with or without a field-gradient, caused significant changes in the expression of immune, stress, and temperature response genes (several heat shock proteins, for instance, appear affected). It is notable that experiments of different durations activated or repressed different genes, although in similar GO groups (Figure 3), and also that these GO groups are similar to the ones found in real microgravity conditions in space [21,23]. Our results indicate that the vertical diamagnetic force (that levitated the flies in the 0 g* tube and enhanced the net effective weight of the flies in the 2 g* tube) had less of an influence on the transcription profile than other effects of the strong magnetic field, which was present in all three tubes (11.5 T in 0 g* and 2 g*, and 16.5 T in 1 g*). Since the differences between the samples in the magnet and samples placed well away from the magnet increased with the duration of the experiment, this suggests that the effects of the magnetic field accumulated with time. This could be explained if the population was less synchronised in "long-term" experiments, and could also be due to different sensitivities of males and females to the magnetic field, *i.e*. affecting to sexual or reproductive parameters.

### C) Magnetic levitation is an alternative to microgravity simulation on Earth allowing isolation of magnetic and gravitational effect

By comparing the samples from the 0 g* and 2 g* arenas (in which there was a strong magnetic field with large field gradient) with samples from the 1 g* arena (strong magnetic field, insignificant gradient), we have identified a number of genes that were affected only by the presence of a strong magnetic field gradient (Figure 3). One such gene, CG6908, was altered *only *by the presence of the large field gradient, i.e. it was unaffected by a magnetic field with no field gradient. Since the field gradient alters the flies' effective weight, we speculate that CG6908 could play a role in a putative signalling cascade involved in the gravity response of *Drosophila*.

Some interesting genes were altered by the magnetic field, with or without the field gradient in our samples, one of which was the Yuri Gagarin gene, a gravity-response gene described previously in *Drosophila *[38]. We also located a number of genes in which ion/metal-related enzymes were encoded (*e.g*. CG4312-RA). These alterations could be related directly to the influence of the magnetic field on the ions [47], or only by the deletion/delocalisation of the ions/metals in the cytosolic or extracellular medium. The gene expression experiments showed that the effect of the magnetic field was comparable to the effect of altered gravity. With careful design of the control experiments, we have demonstrated how the effect of magnetically-altered gravity on gene expression can be distinguished from other effects of the strong magnetic field.

### D) Effect of strong magnetic field on oogenesis

One of the most striking results of this study was an inhibition of oogenesis, or at least its attenuation, by magnetic fields, a fact already observed with weaker and short-lasting magnetic fields [33] and consistent with the gender-related differences in microarray analysis. Several decades ago it was suggested that during the first stages of embryogenesis some electrical potential differences around the embryo may be involved in the origin/maintenance of axis formation, which allows the developmental program to continue [48]. These axes are believed to be created or oriented by the gravity vector (at least originally). Our results suggest that this process is still active in the embryos, and that diamagnetic levitation can partially suppress the generation of the charge distribution at the embryo surface [34]. Additionally, the effect on the phenotype is much more severe when gravity plus charges are simultaneously lost, causing more than 90% of the eggs to fail to complete embryogenesis and metamorphosis to imagoes (Table 2). A similar synergistic effect has been observed already in spaceflight and simulated microgravity experiments under suboptimal environmental conditions [21].

## Conclusions

In summary, we have performed a series of experiments to examine the effects on fruit flies of magnetically-induced weightlessness (0 g*) and simulated hypergravity (2 g*), using exposure times up to 22 days. The data for 0 g* (diamagnetically levitated) conditions show significant similarities with those obtained in related experiments in which a Random Positioning Machine was used to simulate microgravity.

For both 0 g* and 2 g* conditions, a significant delay was observed in the development of the flies from embryo to adult, compared to normal gravity conditions and also significant changes in the expression of immune, stress and temperature response genes. We detected similar but weaker effects on the flies exposed to the strong magnetic field only at the 1 g* condition.

Experiments on gene transcription are sensitive to small variations in environmental conditions. The ability to perform experiments simultaneously under the same conditions of lighting, air temperature, pressure and humidity, enables us to attribute unambiguously the effects we observe in the magnet to the altered effective gravity environment and magnetic field.

The effects of the magnetic field and magnetic field gradient reported here suggest that the transcriptome is finely tuned to the environmental conditions and that relatively small differences in the design of an experiment or the population chosen could lead to different gene expression profiles.

## Authors' contributions

RH carried out RPM experiments, both RPM and magnet sample processing, molecular genetic studies and data analyses and drafted the manuscript. OJL, CED and RM conceived the study, designed the experimental apparatus for the magnet system and carried out the experiments in the magnet. OJL constructed the apparatus. RJAH developed the experimental apparatus with OJL, performed the calculations of the magnetic field and effective gravity, operated and advised on the use and effects of the magnet system, and edited the manuscript. PA, MRD, LE (magnet experiments), FJM and JJWAL (RPM experiment) participated in the study design and coordination and helped to draft the manuscript. All authors, except the late Professor RM, read and approved the final manuscript.

## Supplementary Material

Additional file 1 **Supplementary online material including additional methods and figures S1 to S6**. Click here for file

Additional file 2 Ten minute-long clip of levitating flies in the 0 g* arena (QuickTime format) also available with a Drosophila walking discussion at [27]. Click here for file

Additional file 3 **GEDI analysis files, including each cluster list of probesets and their expression ratio for each condition (zip file)**. Click here for file
