# Supplementary material for: Microgravity simulation by diamagnetic levitation: effects of a strong gradient magnetic field on the transcriptional profile of Drosophila melanogaster
Source: BMC Genomics. 2012 Feb 1;13:52. doi: 10.1186/1471-2164-13-52 (PMC3305489; doi:10.1186/1471-2164-13-52)

SUPPLEMENTARY ONLINE MATERIAL

Microgravity simulation by diamagnetic levitation: effects of a strong gradient magnetic field on the transcriptional profile of *Drosophila melanogaster*

Raul Herranz[1,2*§], Oliver J Larkin[3*], Camelia E Dijkstra[3*], Richard JA Hill[4], Paul Anthony[3], Michael R Davey[3], Laurence Eaves[4], Jack JWA van Loon[5], F Javier Medina[1], Roberto Marco[2]

[1]Centro de Investigaciones Biológicas (CSIC), Ramiro de Maeztu 9, E-28040, Madrid, Spain

[2]Dpto. Bioquímica, Universidad Autónoma de Madrid, Arzobispo Morcillo, E-28009 Madrid, Spain

[3]School of Biosciences, University of Nottingham, Sutton Bonington Campus, Loughborough, LE12 5RD, United Kingdom

[4]School of Physics & Astronomy, University of Nottingham, Nottingham NG7 2RD, UK

[5]Dutch Experiment Support Center, DESC at OCB-ACTA, VU-University and University of Amsterdam, Amsterdam, the Netherlands

Content of this SOM:

1.- Extended version of the **Methods section.**

2.- Additional **Figure S1. The magnet system located at the University of Nottingham.**

3.- Additional **Figure S2. Magnetic field, field gradient & effective gravity within the magnet.**

4.- Additional **Figure S3. Picture of *Drosophila* arena contained within a 25 ml tube, effective gravity within the tube, and CCD and lighting system.**

5.- Additional **Figure S4. Venn diagrams showing the number of genes affected by the short-/medium- /long-term exposure to the magnet positions.**

6.- Additional **Figure S5. Venn diagrams showing the number of genes affected by altered gravity simulation.**

7.- Additional **Figure S6. Virtual pathway including genes affected by the magnet field.**

EXTENDED MATERIALS AND METHODS SECTION

Effective gravity

The effective gravity in diamagnetic levitation experiments has been discussed in detail elsewhere (see, for example, [1-3]). Here, we give the expression for the effective gravity acting on water, for reference. The potential energy of water, per unit mass, in the magnet bore is given by

$$U = gz - \frac{\chi}{2\rho\mu_0}B^2$$

where g =9.8 ms^{-2} is the acceleration due to gravity, $\chi = -9 \times 10^{-6}$ and $\rho = 1000$ kg m^{-3} are the volume magnetic susceptibility (in SI units) and density of water respectively, z is the vertical position in the magnet bore, $\mu_0 = 4\pi \times 10^{-7}$Hm^{-1}, and B is the magnetic field, here measured in Tesla (T), which varies with position inside the bore.

The effective gravity is the gradient of the potential energy

$$\mathbf{\Gamma} = -\nabla U$$

The effective gravity is a vector quantity which, in general, can have both a vertical and a horizontal component, as shown in Figure S3. On the axis of the magnet bore, the horizontal component of the effective gravity is zero and the vertical component is $\Gamma = -\partial U/\partial z$. In this paper, we refer to the effective gravity on the axis of the bore, unless stated otherwise.

Magnetic field

There have been recent reports in the literature that high magnetic fields exert effects on bacteria [4], plants [1, 5-6] and animals [7], and also flies [8]. It is therefore crucial to separate the effects of altered gravity from other effects of the high magnetic field. At the centre of the solenoid (Figure S1), the effective gravity on water is close to 1*g*, despite the presence of a large magnetic field of 16.5 T, since the gradient of the magnetic field is small here. Experiments in the central region of the bore (1*g**), were used to assess the effects of high magnetic fields alone on samples. In the arena located 80 mm above the centre, the effective gravity acting on water within the arena was within 5 percent of zero. In the arena located 80mm below the centre, the effective gravity on water within the arena is within 5 percent of 2*g*. In both 0*g** and 2*g** arenas, the samples are subjected to a magnetic field of 11.5T. In order to differentiate between effects due to the vertical diamagnetic force and other effects of the strong magnetic field, experiments were conducted in the three arenas within the magnet bore (0*g**, 1*g** and 2*g**), simultaneously with an external control in an incubator (1*g* arena) well away from the magnet. The effective gravity on water, at the axis of the bore, is shown in Figure S2C. The variation in effective gravity within the 0*g** arena is shown in Figure S3, top right.

Arenas

Drosophila samples were exposed to the levitation magnet inside 25 ml capacity sample tubes (Sarstedt) with 3 ml of semi-solid yeast-based *Drosophila* food in the bottom of each tube, which also maintains humidity in the arena at close to 100 percent. Pictures of the tubes containing the arenas are shown in Figure S3.

A disk of transparent cellophane, held between two retaining black rubber o-rings was positioned 28mm above base of the tube, and punctured around its perimeter to allow gas exchange. The 10-mm high chamber enclosed by the top of the semi-solid food, the cellophane disk and the walls of the tube, formed the arena (Figure S3, top left). This small region limited the effective gravity to which the flies were exposed (within 5% of 0*g* in the top arena, and within 5% of 2*g* in the bottom arena). A maximum of 40 imagoes or pupa can be contained within each arena. The temperature of the arenas was maintained by passing dry air through a copper heat exchange coil immersed in a thermostatically controlled water bath and then through the magnet bore. A thermocouple temperature data logger (TC08; Pico Technology Ltd., St. Neots, UK) was used to monitor the temperature of the samples. Thermocouples were installed at each of the three positions in the magnet bore, inside the incubator used for controls, and in the laboratory to measure ambient temperature. A variation of less than ± 0.2°C was maintained between the samples in the bore. The temperature fluctuated by less than ± 0.1°C over the duration of the experiments.

Lighting and cameras

Lighting rings, containing 6 LEDs each [OSRAM Opto Semiconductors GmbH, Regensburg, Germany] with a wide dispersion angle of 120º and a luminous intensity of 495mcd (20mA @ 3.6V), were placed to illuminate each arena identically. When required, a photoperiod was controlled using a mechanical timer (12h light/night periods). The chamber within the bore excluded all ambient light.

CCD cameras were used for *in situ* observation of the three arenas within the magnet bore, and also of the controls in the incubator (Figure S3-bottom right). Fire-i™ Digital Board Cameras [Unibrain S.A., Athens, Greece] were stripped of non-essential magnetic components, and the remote camera heads were mounted in rings allowing them to be positioned between the sample tubes within the magnet bore. The cameras were daisy-chained together allowing simultaneous monitoring of all positions.

Drosophila microarray analysis (gene expression)

Drosophila samples were recovered as quickly as possible from the experiment container and distributed in groups of at least 12 male imagoes or 8 female imagoes. Total RNA was extracted from the flies using TRIZOL® reagent, allowing the preparation of at least three biological replicates of each condition. The RNA samples that were recovered with enough quality and quantity after a purification/concentration step with an RNeasy miniKit (Qiagen®) were hybridised using Affymetrix® Drosophila 2.0 whole genome GeneChip arrays (2-4 arrays per condition, except in the medium-term females experiment and 2*g** long-term male condition in which only 1 array was hybridised). Fifty four RNA samples were hybridised with chips and were classified as follows; 45 were magnet experiment chips with at least 2 replicates, 5 were for an RPM control experiment and 4 corresponded to orphan arrays (complete female mid-term experiment + 2*g** male long-term experiment conditions) that are not considered in the global analysis.

The CEL files obtained using this protocol (Table 1) have been submitted to the corresponding public database with the global accession number *E-MEXP-2082.* They were analyzed using GeneSpring GX 10 software using the following methods to find differential expression among conditions:

- The RMA summarization algorithm was used, baseline to median of all samples.
- The quality of the RNA introduced into the chip was evaluated with the 3′/5′ ratio. Male samples in medium and long-term experiments showed an optimum quality in comparison with the short-term samples, but the number of replicates was greater in the short-term experiment, so the results should be of a similar significance in both cases.
- The probe set was filtered on a 20 to 100 percentile values, so 17842 of the 18952 probe sets were present in at least 1 array.
- Differentially-expressed genes were detected using a volcano-plot comparison with a p-value cut-off of 0.05 and a fold difference >1.7 with a parametric test that assumed equal variances (except for the three orphan arrays of female medium-term experiment, in which the fold level was used as unique parameter).

GEDI analysis

A global and integrative analysis was carried out using the “gene expression dynamics inspector” (GEDI) self-organizing maps, with version 2.1 of the software [9]. First, we applied the RMA algorithm for background correction, normalization and expression level summarization of the arrays (see above) using GeneSpring v7.3 software (Agilent Technologies).

Next, we calculated the average signal ratio for the 18921 probe sets that showed an expression above 20th percentile in half of the experimental conditions versus its relative 1g control, and used this value for GEDI analysis. Mosaics of 20 x 16 grid size (average of 59 genes/tile) were obtained using the following settings of the software:

Training iterations:	first phase: 60	second phase: 120
Neighbourhood radius:	first phase: 4.0	second phase: 1.0
Learning factor:	first phase: 0.5	second phase: 0.05
Neighbourhood block size:	first phase: 4	second phase: 2
Conscience:	first phase: 3.0	second phase: 3.0

Random seed: 1

Distance metrics: Euclidean

Linear Initialization Method

References

1. Adair RK: **Comment: Influence of stationary magnetic fields on water relations in lettuce seeds.** *Bioelectromagnetics* 2002, **23:**550; discussion 551-552.
2. Hill RJ, Eaves L: **Vibrations of a diamagnetically levitated water droplet.** *Phys Rev E Stat Nonlin Soft Matter Phys* 2010, **81:**056312.
3. Valles JM, Jr., Maris HJ, Seidel GM, Tang J, Yao W: **Magnetic levitation-based Martian and Lunar gravity simulator.** *Adv Space Res* 2005, **36:**114-118.
4. Tsuchiya K, Okuno K, Ano T, Tanaka K, Takahashi H, Shoda M: **High magnetic field enhances stationary phase-specific transcription activity of Escherichia coli.** *Bioelectrochem Bioenerg* 1999, **48:**383-387.
5. Reina FG, Pascual LA: **Influence of a stationary magnetic field on water relations in lettuce seeds. Part I: theoretical considerations.** *Bioelectromagnetics* 2001, **22:**589-595.
6. Reina FG, Pascual LA, Fundora IA: **Influence of a stationary magnetic field on water relations in lettuce seeds. Part II: experimental results.** *Bioelectromagnetics* 2001, **22:**596-602.
7. Valiron O, Peris L, Rikken G, Schweitzer A, Saoudi Y, Remy C, Job D: **Cellular disorders induced by high magnetic fields.** *J Magn Reson Imaging* 2005, **22:**334-340.
8. Ramirez E, Monteagudo JL, Garcia-Gracia M, Delgado JM: **Oviposition and development of Drosophila modified by magnetic fields.** *Bioelectromagnetics* 1983, **4:**315-326.
9. Eichler GS, Huang S, Ingber DE: **Gene Expression Dynamics Inspector (GEDI): for integrative analysis of expression profiles.** *Bioinformatics* 2003, **19:**2321-2322.

ADDITIONAL FIGURES

Figure S1. The magnet system at the University of Nottingham
The picture shows the cryostat enclosing the magnet, with closed cycle cooling system (turret on top) and water bath used to regulate the bore temperature. The entrance to the 5 cm-diameter open bore is indicated by the arrow.

Magnet bore

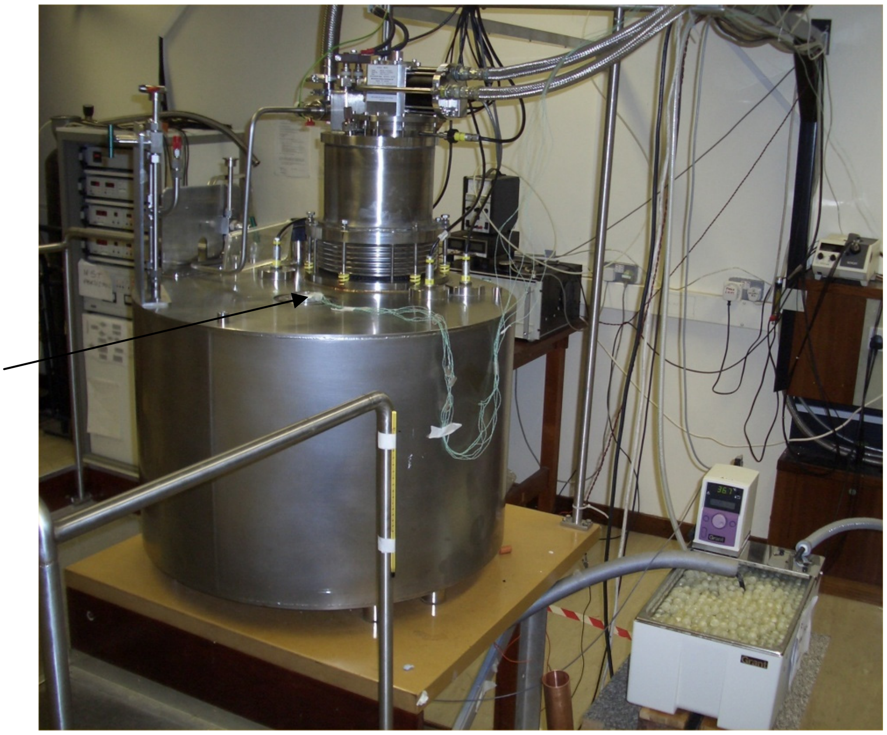

Figure S2. Plots of the magnetic field (A), field gradient (B) and effective gravity (C) on the axis of the magnet bore, as a function of vertical position within the bore, z. The centre of the magnet bore is at $z = 0$ mm. The vertical positions of the 0g*, 1g* and 2g* arenas are indicated by the red lines. Plot C gives the effective gravity acting on water, as a percentage of g.

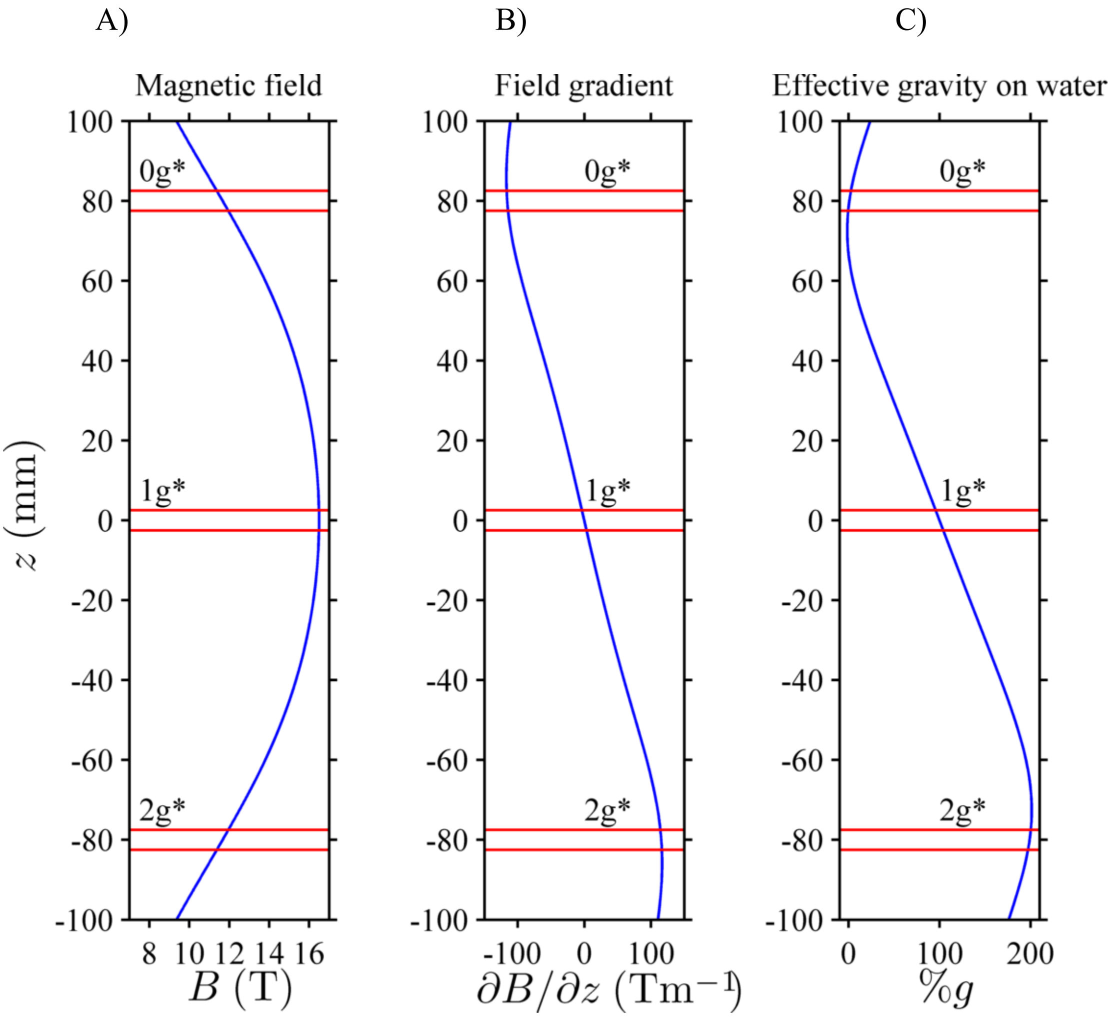

Figure S3. Arena container properties

TOP LEFT) Close-up of one of the arenas contained within a transparent plastic tube 25mm diameter, 50mm tall, viewed from the side. The semi-solid culture medium is visible as the off-white material at the bottom of the tube. The ceiling of the arena is a disk of transparent cellophane, (just visible between the two retaining black rubber o-rings in the image, 25-30mm above the tube's base) punctured around its perimeter to allow gas exchange. **TOP RIGHT)** Contour plot showing the effective gravity acting on pure water within the 25 ml 0*g** sample tube. The contours show the magnitude of the effective gravity as a percentage of 1*g*. The arrows show the direction of the effective gravity. **BELOW LEFT)** Overhead view of arena from *in situ* CCD camera. **BELOW RIGHT)** Picture of tube with CCD camera and LED lighting installed.

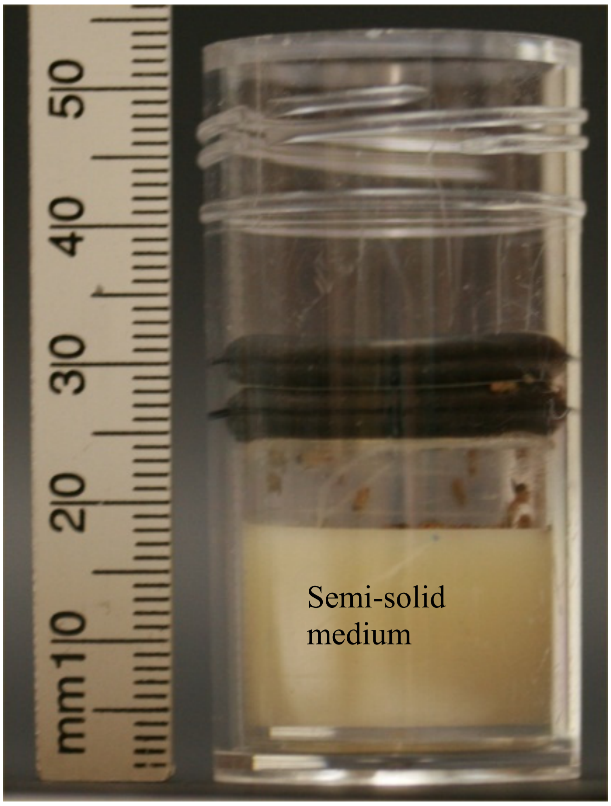

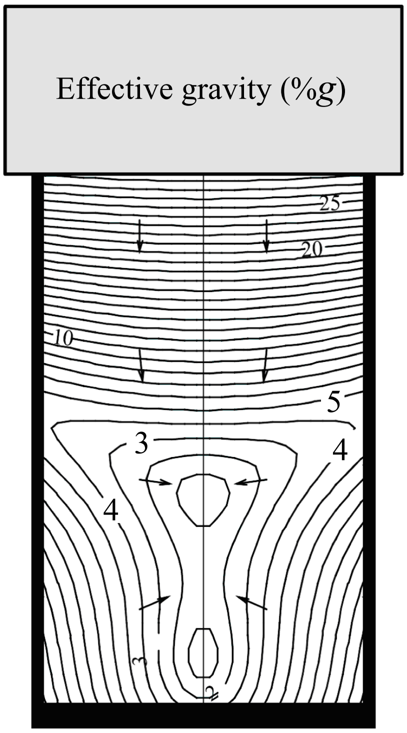

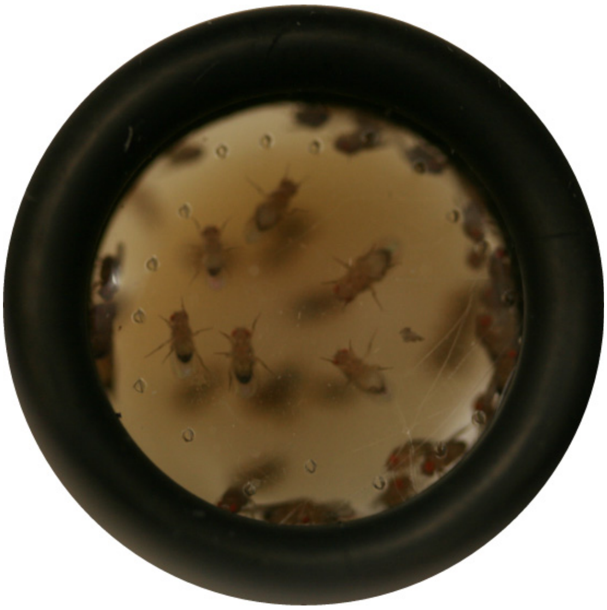

In situ view of arena from CCD camera within magnet bore

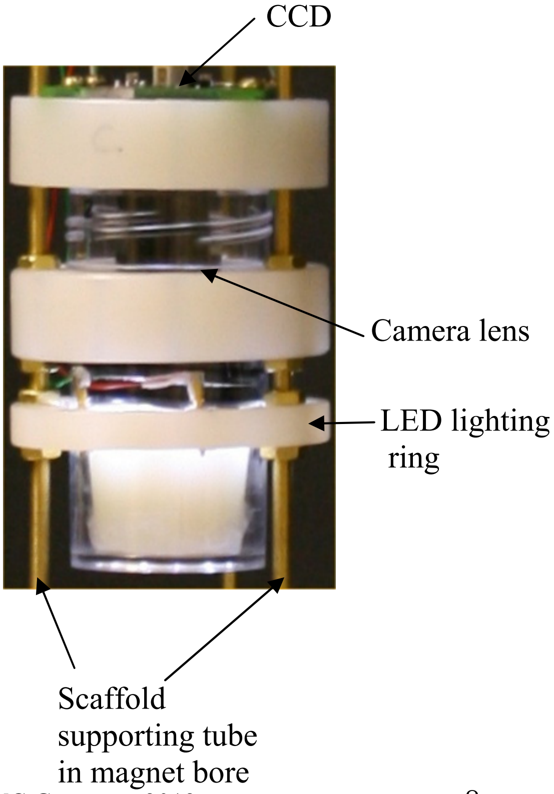

Figure S4. Venn diagrams showing the number of genes affected by the short, medium and long-term exposure to the magnetic field

Numbers indicate probe sets with a fold change in expression >1.7 and a p-value cut-off <0.05. First row, comparison of the different positions in the magnet (0*g**, 1*g**, 2*g**) and controls (14ºC, 19ºC, 24ºC) in short-term experiments. Second row, similar comparisons in medium & long term experiments, including also a comparison of the long-term RPM experiment (grey panel). Third row, comparison among all genes affected in one or at least in 2 experiments in the short, medium and long term experiments in males (left) and females (right) and comparison between males and females pooled results (centre). Note that the results derived from orphan microarrays (indicated with #) have not been used in the global analysis.

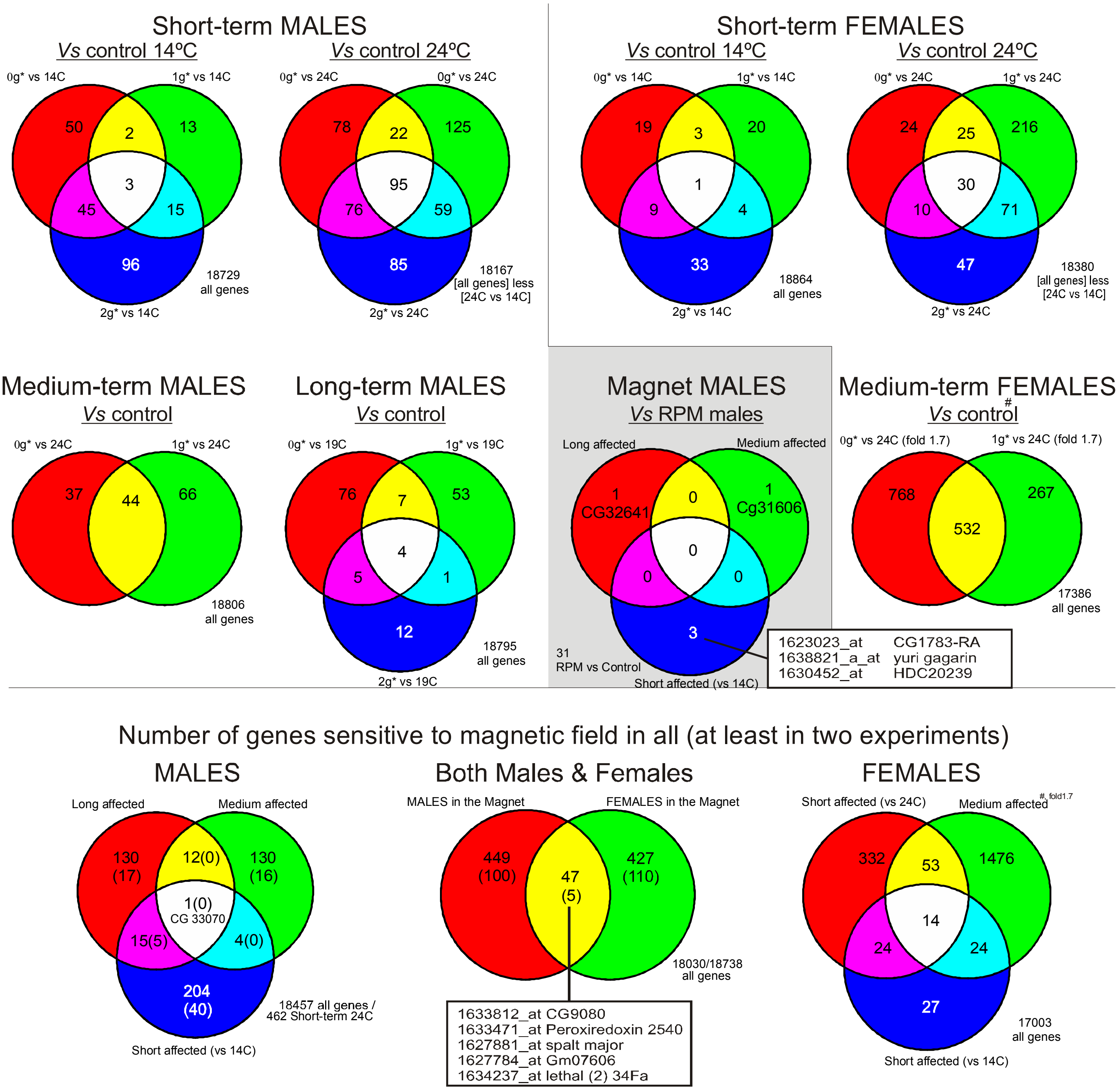

Figure S5. Venn diagrams showing the number of genes affected by altered gravity simulation. Numbers indicate probeset with a fold change in expression >1.7 and a p-value cut-off <0.05. First row, Approach 1 remove magnetic effect subtracting 1*g** effect from 0*g** or 2*g** samples in the different experiments (short-male (SM), short-female (SF), medium (M) and long (L)-term). Grey panel compares all 0*g** (excluding orphan arrays, #) or 2*g** detected genes in previous panels with the 47 magnet affected genes from Figure S4 third row central panel. Several stress/metal/ions related proteins are commonly affected with this approach (square panels). Second row, Approach 2 extract information from some sectors in Figure S4 first and second row panels to locate micro- or hyper-gravity effect and compare these among the different experiments. Third row, comparison among approaches 1 & 2 and RPM affected probesets. Few coincidences are present although some interesting stress-related genes or space-related genes appear. Table bellow indicates the gene ontology groups more relevant in any of the probeset lists obtained from the third row comparisons.

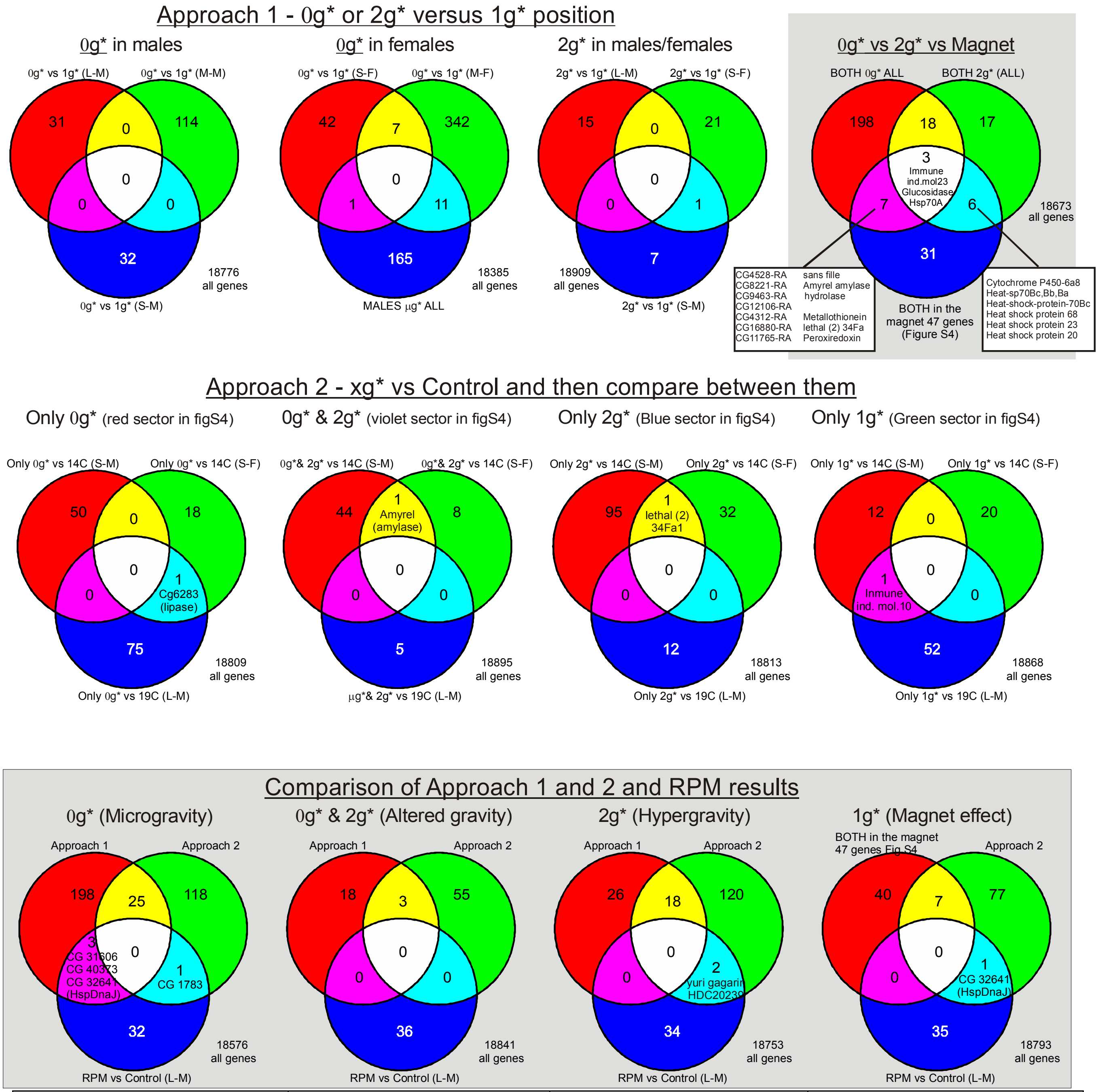

Levitation (simulated microgravity)			Any gravity perturbation			Magnetically induced hypergravity			Magnet effect		
**Approach1**	**Both**	**Approach2**	**Approach1**	**Both**	**Approach2**	**Approach1**	**Both**	**Approach2**	**Approach1**	**Both**	**Approach2**
GO:45087: innate immune response	GO:17000: antibiotic biosynthesis	GO:48731: system development	GO:6952: defense response	NA	GO:7305: vitelline membrane	GO:6629: lipid metabolism	GO:9408: response to heat	GO:7167:enzymelink receptor protein signalling pathway	GO:9408: response to heat	GO:9617: response to bacteria	GO:6959: humoral immune response
GO:5975: carbohydrate metabolism	GO:17144: drug metabolism	GO:7399: nervous system development	GO:9607: response to biotic stimulus	NA	GO:42364: water-soluble vitamin biosynthesis	GO:48252: lauric acid metabolism	GO:6952: defense response	GO:7293: egg chamber formation (sensu Insecta)	GO:9266: response to temperature stimulus	GO:43207: response to ext. biotic stimulus	GO:9613: response to pest, pathogen or parasite
GO:19732: antifungal humoral response	GO:16999: antibiotic metabolism	GO:50830: defense response to Gram+ bacteria	GO:42742: defense response to bacteria	NA	GO:53: argininosuccinate metabolism	GO:8202: steroid metabolism	GO:9607: response to biotic stimulus	GO:30707: ovarian follicle cell devel. (sensu Insecta)	GO:6457: protein folding	GO:9605: response to external stimulus	GO:6955: immune response
GO:6406: mRNA export from nucleus	GO:186: activation of MAPKK activity	GO:6960: antimicrobial humoral response Protostomia	GO:9617: response to bacteria	NA	GO:19627: urea metabolism	GO:6118: electron transport	GO:9266: response to temperature stimulus	GO:7166: cell surface receptor linked signal transduction	GO:6952: defense response	GO:42742: defense response to bacteria	GO:43207: response to external biotic stimulus
GO:50832: defense response to fungi	GO:7256: activation of JNKK activity	GO:50832: defense response to fungi	GO:50829: defense response to Gram-bact.	NA	GO:8628: induction of apoptosis by hormones	GO:44255: cellular lipid metabolism	GO:6457: protein folding	GO:9653: morphogenesis	GO:9607: response to biotic stimulus	GO:9607: response to biotic stimulus	GO:19722: calcium-mediated signalling

Figure S6. Virtual pathway including genes affected only by the magnet field (1*g** versus 1*g* control) with a fold change greater than 2.5 or less than 0.4 (highlighted in fuchsia) are presented in their cytological location and related to other genes than are used as connectors among them. Only 1 order of expansion has been used with all genes, but heat shock proteins, due to their high connectivity, have just the minimum neighbours presented. This pathway has been created using Pathway Studio 6.1 software from Ariadne, Rockville, USA.

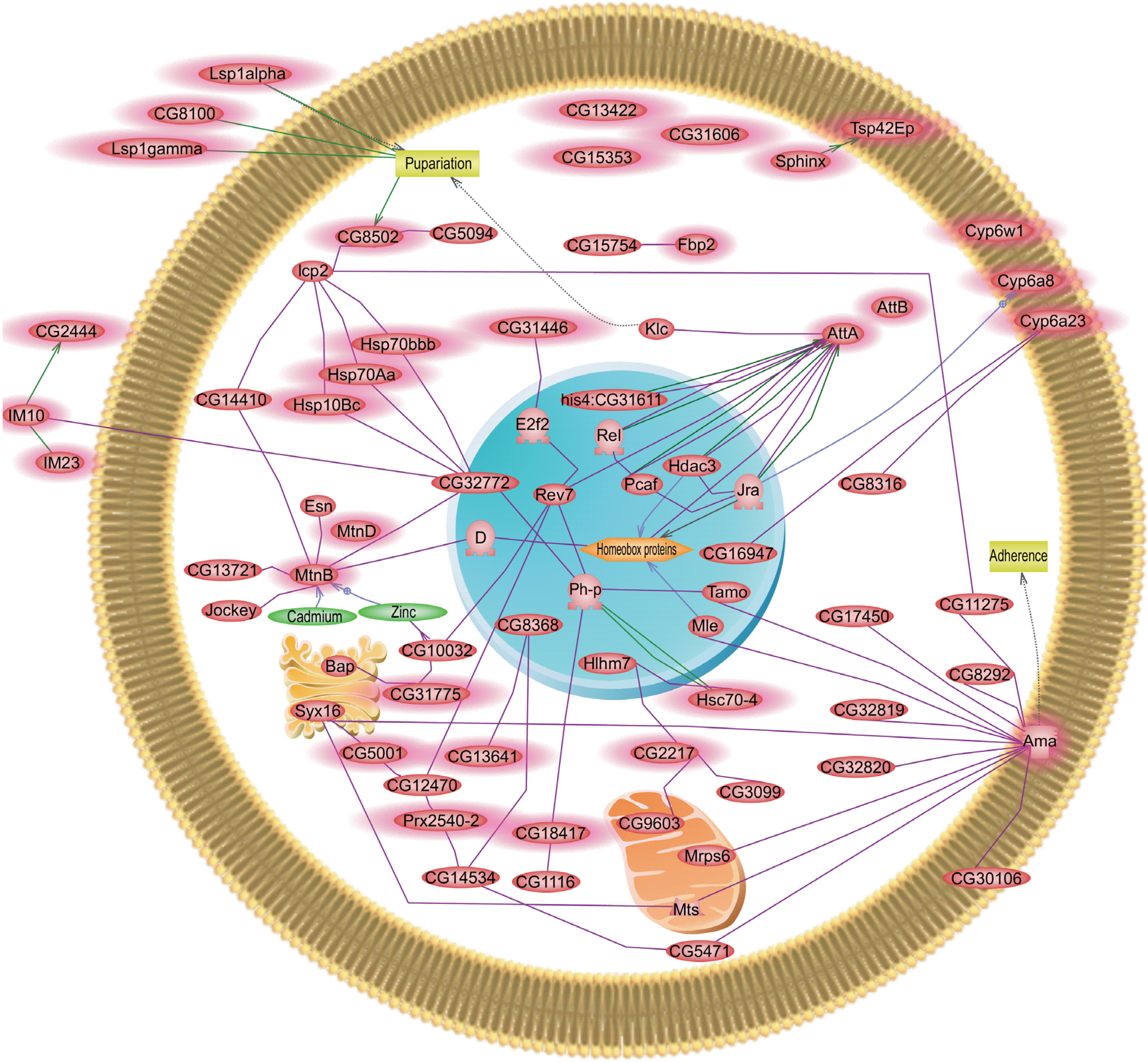

Supplement: Additional file 1 — Supplementary online material including additional methods and figures S1 to S6. [file 1471-2164-13-52-S1.PDF]
