# Supplementary material for: Microgravity simulation by diamagnetic levitation: effects of a strong gradient magnetic field on the transcriptional profile of Drosophila melanogaster
Source: BMC Genomics. 2012 Feb 1;13:52. doi: 10.1186/1471-2164-13-52 (PMC3305489; doi:10.1186/1471-2164-13-52)
Supplement: Additional file 3 — GEDI analysis files, including each cluster list of probesets and their expression ratio for each condition (zip file). [file 1471-2164-13-52-S3.ZIP › Herranz BMCGenomics _ GEDI/0Z_Display.htm]

Magnet 20x16 plus - Drosophila - GEDI MAP 


## Magnet 20x16 plus - Drosophila

Total **24** experiments, **24** data columns, each has **18921** genes.

|  |  |  |  |  |  |  |  |  |  |  |  |  |  |  |  |  |  |  |  |  |  |  |  |  |  |  |  |  |  |  |  |  |
| --- | --- | --- | --- | --- | --- | --- | --- | --- | --- | --- | --- | --- | --- | --- | --- | --- | --- | --- | --- | --- | --- | --- | --- | --- | --- | --- | --- | --- | --- | --- | --- | --- |
| |  |  |  | | --- | --- | --- | | Parameters | Phase 1 | Phase 2 | | Train number: | 60 | 120 | | Neighborhood Radius: | 4 | 1 | | Learning Factor: | 0.5 | 0.05 | | Neighborhood Block Size: | 4 | 2 | | Conscience Factor: | 3.0 | 3.0 | | Grid Size: | 20x16 | | | Similarty Metrics: | Euclidean Distance | | | Init Method: | Linear Initilization | | | Random Seed: | 1 | | |  | Gene Density Map |

|  |  |  |  |  |
| --- | --- | --- | --- | --- |
| LT Mal 0g | LT Mal 1g | LT Mal RPM | MT Mal 0g | MT Mal 1g |
| ST Fem 0g | ST Fem 1g | ST Fem 2g | ST Fem 24 | ST Mal 0g |
| ST Mal 1g | ST Mal 2g | ST Mal 24 | -LT Mal 2g- | -MT Fem 0g- |
| -MT Fem 1g- | LT 0g vs 1g | LT 2g vs 1g | MTm 0g vs 1g | MTf 0g vs 1g |
| STm 0g vs 1g | STm 2g vs 1g | STf 0g vs 1g | STf 2g vs 1g |

|  |  |
| --- | --- |
| Date of Generation: | Thu Dec 03 13:07:47 CET 2009 |
